# Genomic characterization of *Klebsiella pneumoniae* isolates from sepsis patients in Ethiopian tertiary hospitals: a multicenter cross-sectional study

**DOI:** 10.3389/fmicb.2026.1775426

**Published:** 2026-04-28

**Authors:** Melese Hailu Legese, Daniel Asrat, Dejenie Shiferaw Teklu, Ashenafi Alemu Wami, Badrul Hasan, Adane Mihret, Abraham Aseffa, Göte Swedberg

**Affiliations:** 1 Department of Medical Laboratory Sciences, College of Health Sciences, Addis Ababa University, Addis Ababa, Ethiopia; 2 Armauer Hansen Research Institute, Addis Ababa, Ethiopia; 3 Department of Medical Biochemistry and Microbiology, Biomedical Centre, Uppsala University, Uppsala, Sweden; 4 Department of Microbiology, Immunology and Parasitology, College of Health Sciences, Addis Ababa University, Addis Ababa, Ethiopia; 5 Ethiopian Public Health Institute, Addis Ababa, Ethiopia

**Keywords:** carbapenemase genes, extended-spectrum beta-lactamase (ESBL), Ethiopia, *Klebsiella pneumoniae*, sepsis, whole genome sequencing

## Abstract

**Background:**

Treating sepsis caused by high-priority *K. pneumoniae* remains challenging, particularly in low- and middle-income countries.

**Methods:**

A multicenter study was conducted from October 2019 to September 2020 at four hospitals across Ethiopia's central, southern, and northern regions. Blood cultures from 1,416 patients suspected of sepsis were processed, and isolates were identified using MALDI-TOF. Whole-genome sequencing (WGS) was performed on an Illumina HiSeq 2500, and various bioinformatics tools were used for genome analysis and visualization.

**Results:**

A total of 97 *K. pneumonia*e isolates were identified from Tikur Anbessa (*n* = 37), Yekatit (*n* = 30), Hawassa (*n* = 20), and Dessie (*n* = 10) hospitals. Phylogenetically diverse *K. pneumoniae* variants (35 sequence types) were identified, with ST39 (*n* = 15), ST14 (*n* = 11), ST391 (*n* = 10), and ST397 (*n* = 9) being the most common. Almost all isolates (*n* = 94) carried ESBL genes, mainly *bla*_CTX − M−15_, while *bla*_TEM − 1_ (*n* = 75), *bla*_SHV − 11_ (*n* = 43), and *bla*_OXA − 1_ (*n* = 41) were frequently detected, along with other β-lactamases. Carbapenemase genes included *bla*_NDM − 1_ (*n* = 13), *bla*_NDM − 5_ (*n* = 4), and *bla*_OXA − 181_ (*n* = 4). Several additional genes were identified that confer resistance to aminoglycosides (*n* = 70), sulphonamides (*n* = 70), tetracyclines (*n* = 64), trimethoprim (*n* = 54), phenicol (*n* = 40), quinolones (*n* = 37), and macrolides (*n* = 15). Frequent plasmid replicons were Col(pHAD28), Col440I, IncFII(K), IncFIB(K), IncFIB(K)(pCAV1099-114), IncR, and repFIB. Plasmid-borne antimicrobial resistance genes were frequently detected, with IncC exhibiting the broadest spectrum across multiple antibiotic classes. *Yersiniabactin* subtypes *ybt4, ybt8, ybt9, ybt10, ybt13, ybt14, ybt15*, and *ybt16* were identified as virulence genes and were present in 44 of 97 *K. pneumoniae* strains.

**Conclusion:**

*K. pneumoniae* from sepsis patients in Ethiopia showed significant genomic diversity, with widespread ESBL and other plasmid-mediated antimicrobial resistance genes. These findings highlight the urgent need to enhance infection prevention and antimicrobial stewardship nationwide.

## Introduction

*Klebsiella pneumoniae* is a Gram-negative pathogen that causes severe infections (Nguyen et al., 2024) and poses a significant global health burden (Russo and Marr, 2019). It ranks as the second-leading bacterial cause of death (Murray et al., 2022), leading to respiratory, urinary, wound, and bloodstream infections, as well as pneumonia, meningitis, and other diseases (Kochan et al., 2022; Melinte et al., 2025; Cao et al., 2025; Martin and Bachman, 2018; Hussain et al., 2023). *K. pneumoniae* is a leading cause of sepsis (Kochan et al., 2022; Naha et al., 2021): a life-threatening condition resulting from a dysregulated immune response to infection, leading to organ failure and a high mortality rate (Singer et al., 2016). It is an opportunistic pathogen that mainly causes hospital-acquired infections (Melinte et al., 2025), particularly affecting newborns, the elderly, patients with chronic diseases, and immunocompromised individuals (Xu et al., 2025; Sedrakyan et al., 2025; Chaity et al., 2025).

*Klebsiella pneumoniae* has two main pathotypes: classical *K. pneumoniae* (cKp) and hyper-virulent *K. pneumoniae* (hvKp), both found in healthcare settings (Russo and Marr, 2019). cKP primarily causes hospital*-*acquired infections (Russo and Marr, 2019; Kochan et al., 2022), while hvKp strains, with hypermucoviscosity, cause severe community infections (Xu et al., 2025; Sedrakyan et al., 2025). Their virulence varies greatly, affecting morbidity and mortality (Russo and Marr, 2019). Key factors include capsule type, lipopolysaccharide, siderophores, urease, fimbriae, and nutrient acquisition (Xu et al., 2025; Chaity et al., 2025; Das et al., 2022). Siderophores like yersiniabactin (*Ybt*), aerobactin (*Iuc*), and salmochelin (*Iro*) play crucial roles in the virulence of *K. pneumoniae* (Lam et al., 2018; Wisgrill et al., 2019).

The rise of antimicrobial resistance (AMR) threatens public health (Kochan et al., 2022). WHO labels third-generation cephalosporin- and carbapenem-resistant *K. pneumoniae* as a top concern (Organization, 2024). ESBLs like *bla*_CTX − M_, *bla*_TEM_, and *bla*_SHV_ confer resistance to these antibiotics (Lopes et al., 2025; Sewunet et al., 2021). Carbapenem-resistant *K. pneumoniae* develops resistance via carbapenemases like *bla*_KPC_, *bla*_NDM_, and *bla*_OXA_, which inactivate antibiotics such as imipenem, meropenem, and ertapenem (Cao et al., 2025; Martin and Bachman, 2018; Hussain et al., 2023; Xu et al., 2025; Das et al., 2022).

ESBLs and carbapenemase-encoding genes are often carried by mobile genetic elements (MGEs), such as plasmids, transposons, and insertion sequences, which facilitate horizontal gene transfer (Maugeri et al., 2025). Plasmids are vital for acquiring and spreading resistance, as they carry variants such as *bla*_CTX − M_, *bla*_TEM_, *bla*_SHV_ of ESBLs, and *bla*_KPC_, *bla*_NDM_, *bla*_OXA_ of carbapenemases, along with other antimicrobial resistance (AMR) genes (Nguyen et al., 2024; Lopes et al., 2025; Sewunet et al., 2021; Maugeri et al., 2025; Nicitra et al., 2025). Multiple plasmid replicons contribute to the spread of AMR genes (Qamar et al., 2025), such as IncC, IncX, IncFIA, IncFIB, IncFII, IncFIIk, IncH, IncM, IncN, IncL, and Col types (Acman et al., 2022; Rozwandowicz et al., 2018; Cerdeira et al., 2019).

Multi-locus Sequence Typing (MLST) reveals that *K. pneumoniae* is predominantly oligoclonal, with numerous sequence types (STs) identified, such as ST11, ST14, ST15, ST23, ST26, ST48, ST101, ST147, ST149, ST231, ST258, ST627, and ST977 (Naha et al., 2021; Chaity et al., 2025; Rojas et al., 2017; Shankar et al., 2019). Strains from these different STs exhibit variation in virulence and antibiotic resistance, with some specific to certain regions and others associated with outbreaks (Chaity et al., 2025). The spread of multidrug-resistant bacteria is complicating healthcare delivery in low- and middle-income countries (LMICs; Sands et al., 2021), where the burden is believed to be substantial (Lewnard et al., 2024).

Although some studies have reported the burden of AMR (Sewunet et al., 2021; Tufa et al., 2022; Gebremeskel et al., 2023), Ethiopia still lacks comprehensive genomic data on circulating *K. pneumoniae* sequence types, AMR genes, plasmid types, virulence genes, and broader genome features. This information is crucial for effective infection management, prevention, control, and antimicrobial stewardship. Therefore, the aim of this multicenter cross-sectional study was to perform genomic characterization of *K. pneumoniae* isolates from sepsis patients at Ethiopian tertiary hospitals in the central, northern, and southern regions.

## Materials and methods

### Study design, identification, and characterization of *K. pneumoniae* isolates

A prospective multicenter cross-sectional study was conducted at four Ethiopian tertiary hospitals from October 2019 to September 2020. Tertiary hospitals serving large populations, either with their own microbiology laboratories or linked to nearby government regional microbiology laboratories, were purposively selected from different regions of the country. These hospitals included Tikur Anbessa Specialized Hospital (TASH) and Yekatit 12 Specialized Hospital Medical College (Y12HMC) in the central region, Hawassa University Comprehensive Specialized Hospital (HUCSH) in the south, and Dessie Comprehensive Specialized Hospital (DCSH) in the north. During the study, 97 *K. pneumoniae* isolates were identified from patients investigated for sepsis at the four tertiary hospitals. These isolates were initially identified and characterized at the species level using traditional biochemical tests, including indole, urea, citrate, triple sugar iron, motility, and lysine decarboxylase. Malonate was added to the biochemical panel to distinguish *K. pneumoniae* from other *Klebsiella* species. All isolates initially identified by these biochemical methods were later confirmed using MALDI-TOF MS (matrix-assisted laser desorption/ionization time-of-flight mass spectrometry). Antimicrobial testing was performed using disk diffusion, according to the standardized table published by the Clinical and Laboratory Standards Institute (Institute, 2020). Details of each hospital and patient enrolment are provided in the previous work (Legese et al., 2022).

### DNA extraction and whole genome sequencing (WGS)

Genomic DNA was extracted from overnight cultures of each *K. pneumoniae* isolate using the QIAamp DNA Mini Kit (Qiagen, Germany) following the manufacturer's instructions. The DNA extraction process was based on selecting 2 to 5 pure colonies grown on cystine-lactose-electrolyte-deficient agar at 37 °C for 24 h under aerobic conditions. DNA quantity and quality was assessed with a Qubit Fluorometer (Thermo Fisher Scientific, MA, USA). All DNA samples were stored at −20 °C until used for whole-genome sequencing. Libraries for sequencing were prepared using the Nextera XT DNA Library Preparation Kit (Illumina) and sequenced on the Illumina HiSeq 2500 platform, yielding 2 × 150-bp paired-end reads.

### Bioinformatics analysis

#### Quality control and contamination screening

Raw sequencing reads were thoroughly evaluated with FastQC v0.11.9 (Andrews, 2010), and summary reports were compiled using MultiQC v1.32 (Ewels et al., 2016). Quality trimming and adapter removal were carried out with fastp v0.20.1 (Chen et al., 2018) using default settings. Contamination screening employed ConFindr v0.8.2 (Low et al., 2019) to confirm strain purity. All reads passed quality checks, were free of cross-contamination, and proceeded to downstream analyses.

### Species identification, molecular typing, and reference selection

Species identification from whole-genome data was carried out using Bactinspector v0.1.3 (Underwood, 2020), which also identified the closest reference genome for alignment. Multi-locus sequence typing (MLST) was performed with both the standalone MLST tool (v2.23.0; Seemann, 2018) and Kleborate v3.2.4 (Lam et al., 2021), applying the Achtman scheme to determine sequence types (STs) from allelic profiles. The reference genome selected by Bactinspector (NZ_CP009461.1) was used for variant calling and phylogenetic analyses. Capsule (K) and lipopolysaccharide (O) locus typing were done using Kaptive (v3.0; Lam et al., 2022) to identify capsular and O-antigen biosynthesis loci, with *wzi* allele typing performed to improve K-locus predictions and capsular typing accuracy. The presence of capsular polysaccharide (K) and lipopolysaccharide (O) loci was confirmed using Kaptive v0.7.3 (Stanton et al., 2025).

### De novo genome assembly

Genome assembly was performed using the Shovill pipeline (v1.1.0; Seemann, 2016) with the SKESA assembler, employing default parameters for optimal contig generation. The quality of the assembled genomes was evaluated using QUAST (v5.2.0; Gurevich et al., 2013) to assess assembly metrics such as the number of contigs, total assembly length, GC content, N50, and L50. These assessments were conducted to ensure the completeness, contiguity, and adequacy of coverage of the reconstructed genomes for downstream analyses.

### Variant calling and core genome alignment

Core genome single-nucleotide polymorphisms (SNPs) were identified using Snippy v4.6.0 (Seemann, 2020) with default parameters. Paired-end reads were aligned to the reference genome (NZ_CP009461.1), and variants were called with FreeBayes (Garrison and Marth, 2012), requiring a minimum mapping quality of 60, base quality of 13, and at least 10X coverage. The core genome alignment was produced with snippy-core, excluding recombinant regions and positions with missing data.

### Phylogenetic construction and analysis

A core-genome phylogenetic tree was generated to examine the genetic relationships among the isolates. The maximum-likelihood phylogeny was reconstructed from the core genome SNP alignment using IQ-TREE v3.0.1 (Minh et al., 2020). Model selection, performed with ModelFinder (Kalyaanamoorthy et al., 2017), identified the GTR+G model—General Time Reversible with Gamma-distributed rate heterogeneity—as the best fit based on the Bayesian Information Criterion. Branch support was assessed using 1,000 ultrafast bootstrap (Hoang et al., 2018) replicates. For interactive analysis, the phylogenetic trees were visualized and annotated with iTOL v7.2.2 (Letunic and Bork, 2021). Maximum-likelihood phylogenies of the 97 *K. pneumoniae* isolates were reconstructed using IQ-TREE v3.0.1 under the GTR+G substitution model, with branch support assessed by 1,000 ultrafast bootstrap replicates. The tree was annotated in iTOL v7.2.2 (Letunic and Bork, 2021) with sequence type (ST), hospital, ward, yersiniabactin virulence loci, capsule (K-locus), O-locus, antimicrobial resistance (AMR) classes, and plasmid replicon markers.

### Minimum spanning tree construction

To examine the genetic relationships among *K. pneumoniae* isolates, we constructed minimum spanning trees (MSTs) from Multilocus Sequence Typing (MLST) profiles. Allelic profiles from seven housekeeping genes: *gapA, infB, mdh, pgi, phoE, rpoB*, and *tonB*, were used to calculate pairwise allelic distances between sequence types (STs). The MSTs were generated using the NetworkX library in Python, with Kruskal's algorithm to identify the minimum spanning tree.

### Identification of AMR genes, virulence genes, and plasmid replicons

NCBI's AMRFinderPlus v4.0.23 (Feldgarden et al., 2021), using the comprehensive 2025-07-16.1 database, was employed to detect antimicrobial resistance determinants in all assembled genomes. Similarly, virulence genes were also assessed using the NCBI's AMRFinderPlus v4.0.23 platform (Feldgarden et al., 2021). Our analysis used organism-specific profiling of *K. pneumoniae* to detect acquired resistance genes via translated nucleotide searches (tBLASTn) and chromosomal point mutations. Plasmid replicon types were identified with PlasmidFinder v2.1.6 (Carattoli et al., 2014) using default settings against the *Enterobacteriaceae* plasmid database v2.2.0. The results from AMRFinderPlus and PlasmidFinder were combined with phylogenetic data and metadata for comparative analysis. Custom Python scripts (v3.8) were created to identify plasmid replicon types harboring AMR genes, matching data based on sample identifiers and contig names to locate contigs where plasmid replicons and AMR genes co-occur.

### Statistical analysis

The metadata was compiled with Microsoft Office Excel, and a Python script was used for analysis. Descriptive statistics, including count, mean, percentages, frequency, and standard deviation, were calculated. Frequencies of antimicrobial resistance, plasmid replicons, and virulence genes were also assessed.

#### Ethical approval

This study received approval from the Department of Microbiology, Immunology, and Parasitology Ethical Review Committee (DEREC/18/19/01-H) and the Institutional Review Board (AAUMF 01–008) at the College of Health Science, Addis Ababa University. It also gained approval from the AHRI/ALERT Ethics Review Committee (protocol number: P050/18) of the Armauer Hansen Research Institute, as well as the National Ethical Review Committee (Ref No. MoSHE//RD/14.1/690/19).

## Results

A total of 97 *K. pneumoniae* isolates were identified from 1,416 patients across four hospitals: Tikur Anbessa Specialized Hospital (*N* = 37), Yekatit 12 Hospital Medical College (*N* = 30), Hawassa University Comprehensive Specialized Hospital (*N* = 20), and Dessie Comprehensive Specialized Hospital (*N* = 10; Table 1). Most of the *K. pneumoniae* isolates were detected in neonatal intensive care (NICU; *n* = 49) and pediatrics (*n* = 33) units, indicating that neonates and children are more affected by *K. pneumoniae-*related sepsis. Most patients (*n* = 62) experienced a fever, whereas some (*n* = 17) had hypothermia. The majority (*n* = 53) had underlying diseases, and a subset (*n* = 54) reported being referred from other healthcare facilities (Table 1). All patients were hospitalized at each respective facility. All identified *K. pneumoniae* strains were analyzed using whole-genome sequencing to examine their genomic features.

**Table 1:** Demographic and clinical characteristics of sepsis patients with *K. pneumoniae* identified at four Ethiopian Tertiary Hospitals.

Demographic and clinical char acteristics		Overall frequency *n*(%)	DCSH *n*(%)	HUCSH *n*(%)	TASH *n*(%)	Y12HMC *n*(%)
Sex	Male	57(58.8)	7(70)	15(75)	23(62.2)	12(40)
	Female	40(41.2)	3(30)	5(25)	14(37.8)	18(60)
Age category	< 30 days	49(50.5)	10(100)	11(55)	7(18.9)	21(70)
	30 days to 1 year	14(14.4)	–	2(10)	9(24.3)	3(10)
	>1 to < 5 years	10(10.3)	–	3(15)	6(16.2)	1(3.3)
	>5 to < 18 years	11(11.3)	–	3(15)	7(18.9)	1(3.3)
	>18 years	13(13.4)	–	1(5)	8(21.6)	4(13.3)
Ward	EOPD	2(2.1)	–	1(5)	1(2.7)	–
	ICU	5(5.2)	–	–	5(13.5)	–
	Medical ward	9(9.3)	–	–	4(10.8)	5(16.7)
	NICU	48(49.5)	10(100)	11(55)	6(16.2)	21(70)
	Pediatrics	32(33)	–	8(40)	20(54.1)	4(13.3)
	Surgical ward	1(1)	–	–	1(2.7)	–
Hospitalization status	Inpatient	97(100)	10(100)	20(100)	37(100)	30(100)
Hospital stay duration category	1 week	49(50.5)	9(90)	11(55)	5(13.5)	24(80)
	2 weeks	16(16.5)	1(10)	6(30)	6(16.2)	3(10)
	3 weeks	11(11.3)	–	2(10)	8(21.6)	1(3.3)
	4 or more weeks	21(21.6)	–	1(5)	18(48.6)	2(6.7)
Underlying diseases	Yes	53(54.6)	–	9(45)	35(94.6)	9(30)
Referral patient	Yes	54(55.7)	4(40)	11(55)	30(81.1)	9(30)
Previous hospitalization	Yes	17(17.5)	–	5(25)	11(29.7)	1(3.3)
Prior antibiotics use	Yes	35(36.1)	3(30)	11(55)	21(56.8)	–
Fever	Yes	62(63.9)	10(100)	13(65)	27(73)	12(40)
Duration of fever category	3 days or less	62(63.9)	6(60)	15(75)	17(45.9)	24(80)
	4–6 days	18(18.6)	4(40)	5(25)	6(16.2)	3(10)
	7 or more days	17(17.5)	–	–	14(37.8)	3(10)
Hypothermia	Yes	17(17.5)	–	2(10)	1(2.7)	14(46.7)
Tachypnea	Yes	43(44.3)	10(100)	13(65)	5(13.5)	15(50)
Tachycardia	Yes	36(37.1)	6(60)	12(60)	6(16.2)	12(40)
Respiratory distress	Yes	43(44.3)	10(100)	9(45)	8(21.6)	16(53.3)
Apnea	Yes	9(9.3)	1(10)	2(10)	2(5.4)	4(13.3)
Vomiting	Yes	35(36.1)	1(10)	7(35)	15(40.5)	12(40)
Irritability	Yes	20(20.6)	1(10)	2(10)	–	17(56.7)
Hypotension	Yes	2(21)	–	2(10)	–	–
Cough	Yes	21(21.6)	–	5(25)	10(27)	6(20)
Prostration	Yes	18(18.6)	–	–	–	18(60)
Skin rash	Yes	5(5.2)	1(10)	1(5)	1(2.7)	2(6.7)
Bleeding tendency	Yes	9(9.3)	–	1(5)	5(13.5)	3(10)
Lethargy	Yes	11(11.3)	2(20)	4(20)	1(2.7)	4(13.3)
Seizure/convulsion	Yes	5(5.2)	2(20)	1(5)	1(2.7)	1(3.3)
Body weakness	Yes	22(22.7)	–	–	1(2.7)	21(70)

TASH, Tikur Anbessa Specialized Hospital; Y12HMC, Yekatit 12 Specialized Hospital Medical College; DCSH, Dessie Comprehensive Specialized Hospital, HUCSH, Hawassa University Comprehensive Specialized Hospital; NICU, neonatal intensive care unit; EOPD, emergency outpatient department; ICU, intensive care unit; – 0(0).

### Antimicrobial resistance patterns of *K. pneumoniae*

**Figure 1:**
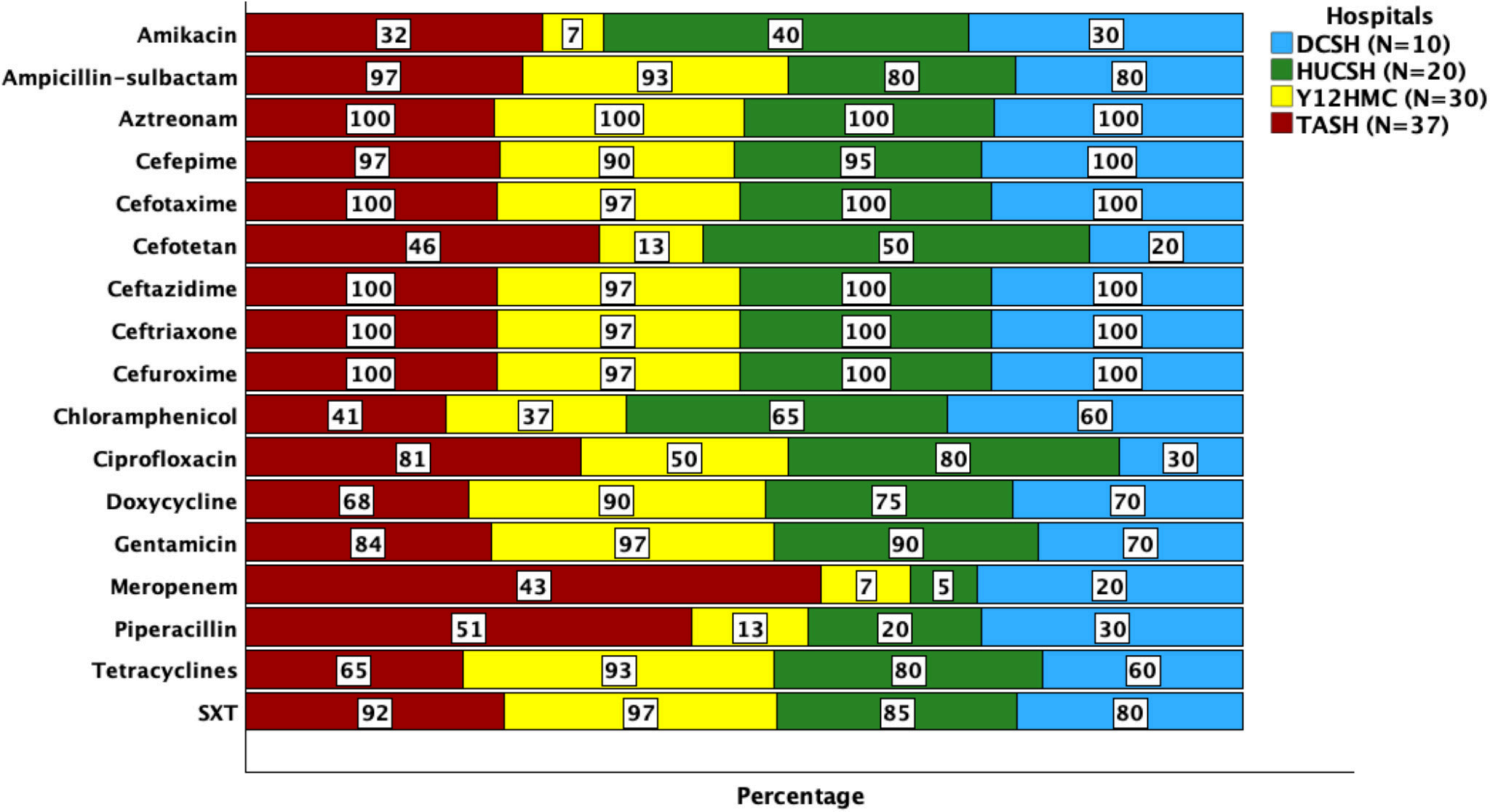
Percentage of antimicrobial-resistant *K. pneumoniae* identified at four Ethiopian Tertiary Hospitals. Note: the numbers indicate the percentage of *K. pneumoniae* isolates resistant to the specified agents within the hospital. TASH, Tikur Anbessa Specialized Hospital; Y12HMC, Yekatit 12 Specialized Hospital Medical College; DRH, Dessie Referral Hospital, HUCSH, Hawassa University Comprehensive Specialized Hospital.

Almost all (99%, *n* = 96) *K. pneumoniae* isolates were resistant to third-generation cephalosporins, and 100% were resistant to aztreonam (Figure 1 and Table 2). Many of them also showed resistance to trimethoprim-sulfamethoxazole, tetracyclines, gentamicin, and ciprofloxacin. Only meropenem and amikacin demonstrated relative effectiveness in treating *K. pneumoniae* (Table 2). Meropenem had the highest resistance rate at TASH 43%(*n* = 16/37), whereas HUCSH and Y12HMC had low resistance rates 5%(*n* = 1/20) and 7%(*n* = 2/30).

**Table 2:** Antimicrobial resistance genes carried by *K. pneumoniae* isolates from sepsis patients at four Ethiopian Tertiary Hospitals.

Antibiotic class	Antibiotic	Overall resistance frequency *n*(%)	Resistance genes	AMR Gene	Overall (*N* = 97) *n*(%)	DCSH (*N* = 10) *n*(%)	HUCSH (*N* = 20) *n*(%)	TASH (*N* = 37) *n*(%)	Y12HMC (*N* = 30) *n*(%)
Beta-lactam	Aztreonam	97(100)	Beta-lactamase	*bla*_CTX − M−15_	94(97)	10(100)	20(100)	35(95)	29(97)
	Cefotaxime	96(99)		*bla*_TEM − 1_	75(77)	8(80)	16(80)	26(70)	25(83)
	Ceftazidime	96(99)		*bla*_SHV − 11_	43(44)	2(20)	12(60)	15(41)	14(47)
	Ceftriaxone	96(99)		*bla*_OXA − 1_	41(42)	1(10)	14(70)	13(35)	13(43)
	Cefuroxime	96(99)		*bla*_SHV − 1_	22(23)	–	5(25)	8(22)	9(30)
	Cefepime	92(94.8)		*bla*_SHV − 28_	13(13)	5(50)	1(5)	6(16)	1(3)
	Cefotetan	33(34)		*bla*_NDM − 1_	13(13)	2(20)	1(5)	8(22)	2(7)
	Piperacillin	30(30.9)		*bla*_SCO − 1_	8(8)	1(10)	–	2(5)	5(17)
	Meropenem	21(21.6)		*bla*_SCO_	7(7)	–	–	2(5)	5(17)
				*bla*_CMY − 6_	7(7)	2(20)	–	5(14)	–
				*ompK35-E132K*	7(7)	–	2(10)	5(14)	–
				*bla*_NDM − 5_	4(4)	–	–	4(11)	–
	Ampicillin-sulbactam	88(90.7)		*bla*_OXA − 181_	4(4)	–	–	4(11)	–
Aminoglycoside	Amikacin	25(25.8)	Aminoglycoside resistance genes	*aph(6)-Id*	70(72)	8(80)	17(85)	22(60)	23(77)
				*aph(3″)-Ib*	70(72)	8(80)	17(85)	22(60)	23(77)
	Gentamicin	85(87.6)		*aac(3)-IIe*	66(68)	3(30)	16(80)	19(51)	28(93)
				*aadA1*	26(27)	–	7(35)	9(24)	10(33)
				*aac(3)-IId*	16(17)	5(50)	5(25)	5(14)	1(3)
				*aadA16*	14(14)	–	2(10)	6(16)	6(20)
				*aph(3′)-Ia*	11(11)	1(10)	2(10)	8(22)	–
				*aadA2*	11(11)	–	3(15)	8(22)	–
Phenicol	Chloramphenicol	45(46.4)	Phenicol resistance genes	*catB3*	40(41)	1(10)	14(70)	13(35)	12(40)
				*catA2*	19(20)	6(60)	6(30)	5(14)	2(7)
				*catA1*	14(14)	1(10)	2(10)	2(5)	9(30)
				*cmlA5*	10(10)	–	4(20)	6(16)	–
Quinolone	Ciprofloxacin	64(66)		*qnrB1*	37(38)	2(20)	12(60)	12(32)	11(37)
				*qnrS1*	15(16)	2(20)	2(10)	9(24)	2(7)
				*parC_S80I*	10(10)	–	2(10)	6(16)	2(7)
Aminoglycoside/fluroquinolones resistance gene				*aac(6′)-Ib-cr5*	53(55)	1(10)	14(70)	19(51)	19(63)
Folate pathway inhibitor	Trimethoprim-sulfamethoxazole	88(90.7)	Trimethoprim resistance gene [dihydrofolate reductase (drf)]	*dfrA14*	54(56)	9(90)	15(75)	16(43)	14(47)
				*dfrA50*	17(18)	5(50)	1(5)	6(16)	5(17)
				*dfrA27*	14(14)	–	2(10)	6(16)	6(20)
				*dfrA15*	14(14)	–	2(10)	2(5)	10(33)
				*dfrA7*	12(12)	1(10)	2(10)	4(11)	5(17)
			Sulfonamide resistance gene	*sul2*	70(72)	8(80)	18(90)	21(57)	23(77)
				*sul1*	53(55)	4(40)	8(40)	25(68)	16(53)
Tetracycline	Tetracyclines	74(76.3)	Tetracyclines resistance gene	*tet(A)*	64(66)	3(30)	16(80)	19(52)	26(87)
	Doxycycline	74(76.3)		*tet(D)*	9(9)	5(50)	1(5)	2(5)	1(3)
Phenicol/quinolone 89(92)				*oqxA*	89(92)	10(100)	19(95)	32(87)	28(93)
				*oqxB*	25(26)	7(70)	2(10)	14(38)	2(7)
				*oqxB19*	23(24)	–	5(25)	5(14)	13(43)
				*oqxB32*	18(19)	1(10)	7(35)	4(11)	6(20)
				*oqxB25*	10(10)	1(10)	3(15)	5(14)	1(3)
Efflux			Multidrug efflux	*emrD*	97(100)	10(100)	20(100)	37(100)	30(100)
Bleomycin				*ble*	17(18)	17(18)	2(20)	1(5)	12(32)
Fosfomycin				*fosA*	87(90)	8(80)	16(80)	34(92)	29(97)
Rifamycin				*arr-3*	15(16)	–	3(15)	6(16)	6(20)
Macrolide				*mph(A)*	15(16)	–	2(10)	5(14)	8(27)

TASH, Tikur Anbessa Specialized Hospital; Y12HMC, Yekatit 12 Specialized Hospital Medical College; DCSH, Dessie Comprehensive Specialized Hospital, HUCSH-Hawassa University Comprehensive Specialized Hospital; – 0(0); Bleomycin, Fosfomycin, Rifamycin, and Macrolide were not tested for *K. pneumoniae* phenotypically.

Other infrequently identified AMR genes not included in the table above were: gyrA_S83I (n = 6), oqxB5 (n = 6); bla_OXA − 10_ (n = 6); fosA10 (n = 6); oqxB14 (n = 5); dfrA30 (n = 5); oqxB20 (n = 5); bla_SHV − 60_ (n = 5); ere(A) (n = 5); bla_SHV − 27_ (n = 4); gyrA_S83Y (n = 4); gyrA_D87A (n = 4); oqxA10 (n = 4); bla_SHV − 187_ (4); ompK36_D135DGD (n = 4); bla_SHV − 71_ (3); qnrB6 (n = 3); fosA5 (n = 3); rmtB1 (n = 3); qnrB4 (n = 2); aac(6′)-Ib (n = 2); bla_DHA − 1_ (n = 2); bla_SHV − 119_ (n = 2); dfrA16 (n = 2); blaOXA (n = 2); bla_DHA − 1_ (n = 2); arr (n = 2); aac(3)-IIg (n = 1); aac(6′)-IIc (n = 1); bla_SHV − 110_ (n = 1); floR2 (n = 1); bla_SHV_C − 112A_ (n = 1); dfrA1 (n = 1); dfrA19 (n = 1); dfrA5 (n = 1); fosA7 (n = 1); mcr-9.1 (n = 1); ompK35_W230STOP (n = 1); fosA9 (n = 1); qnrB (n = 1); oqxB27 (n = 1); ramR_A19V (n = 1); tet(G) (n = 1).

### Antimicrobial resistance genes carried by *K. pnuemoniae*

All 97 *K. pneumoniae* isolates were found to carry at least one resistance gene across different antibiotic classes: 94 were resistant to β-lactams, 70 to aminoglycosides, 70 to sulfonamides, 64 to tetracyclines, 54 to trimethoprim, 40 to phenicols, 37 to quinolones, 15 to macrolides, and 15 to rifampicin (Table 2). *bla*_CTX − M−15_ (*n* = 94), *oqxA* (*n* = 90), *fosA* (*n* = 87), *bla*TEM_−1*B*_ (*n* = 77), *aph(6)-Id* (*n* = 70), *aph(3*″*)-Ib* (*n* = 70), *sul2* (*n* = 70), *aac(3)-IIe* (*n* = 66) and *tet(A)* (*n* = 64) were the most common AMR genes detected (Table 2). *bla*_CTX − M−15_ (*n* = 94) was the most widely disseminated ESBL gene across the four hospitals. *bla*_TEM − 1_, detected in 75% of isolates (*n* = 77/97), was another highly prevalent β-lactamase that can inactivate several β-lactam antibiotics other than extended-spectrum cephalosporins (Table 2). Among all *K. pneumoniae* isolates, 13 *bla*_NDM − 1_ cases were detected across all hospitals, whereas 4 cases of concurrent *bla*_NDM − 5_ and *bla*_OXA − 181_ were identified at TASH. Colistin resistance was detected in only one isolate (Table 2). All *K. pneumoniae* isolates (*n* = 97) carried *emrD*, an efflux pump gene, which may contribute to multidrug resistance by actively pumping out antibiotics.

### Co-occurrence of antimicrobial resistance genes

**Figure 2:**
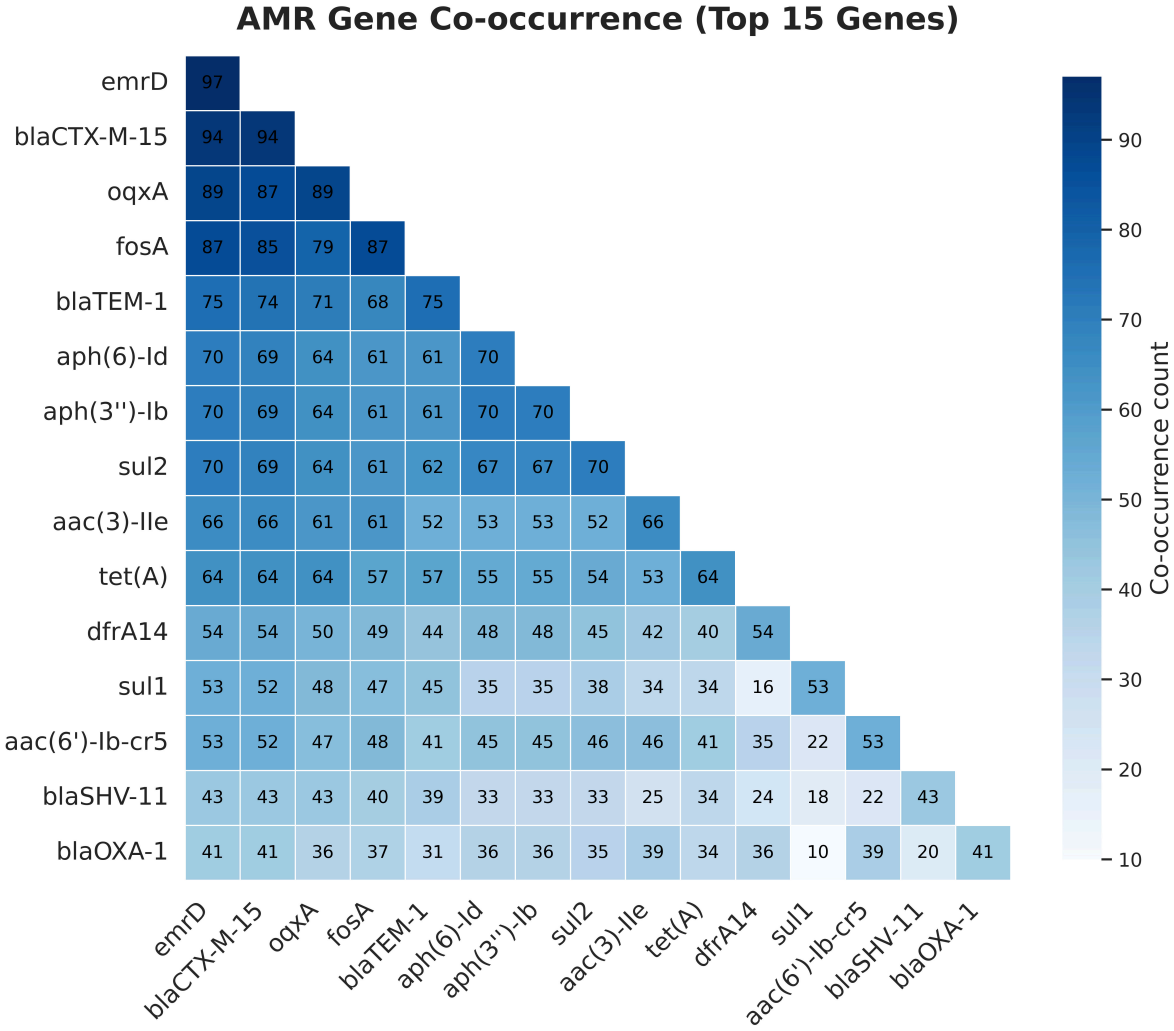
Co-occurrence of the most frequent antimicrobial resistance genes carried by *K. pnuemoniae* identified from sepsis patients at four Ethiopian Tertiary Hospitals.

AMR gene co-occurrence was observed at all study sites (Figure 2), with the most common combination involving ESBLs and other AMR genes associated with different antibiotic classes. The most frequent co-occurrences were *bla*_CTX − M−15_ and *oqxA* (*n* = 87), followed by *bla*_CTX − M−15_ *and fosA* (*n* = 85), and *bla*_CTX − M−15_ and *bla*_TEM − 1B_ (*n* = 74). Other common co-occurrences of *bla*_CTX − M−15_ included *aph(6)-Id* (*n* = 69), *aph(3*″*)-Ib* (*n* = 69), *sul2* (*n* = 69), and *aac(3)-IIe* (*n* = 66), which limit treatment options (Figure 2). Conversely, *bla*_OXA − 1_ co-occurring with other common AMR genes was infrequent (Figure 2).

### Hospital-based clustering and distribution of antimicrobial resistance genes

**Figure 3:**
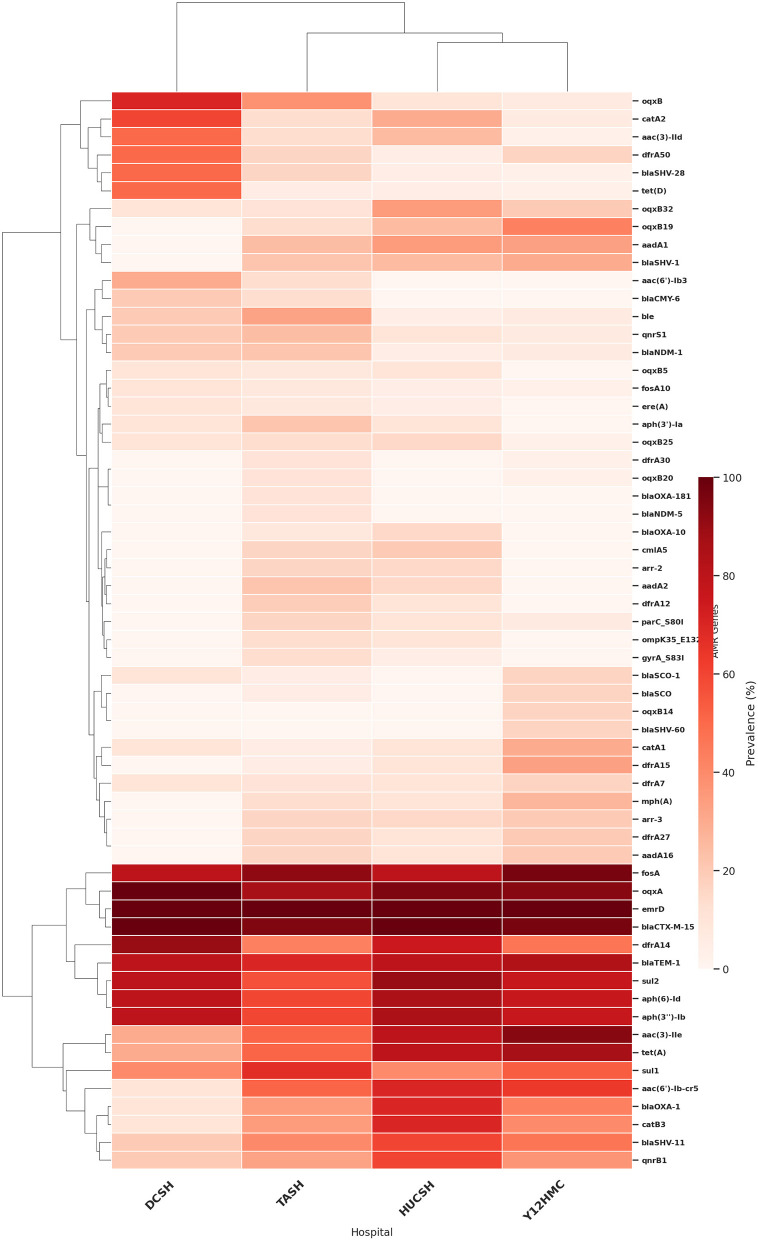
Hierarchical clustering heatmap of the prevalence of antimicrobial resistance genes carried by *K. pneumoniae* isolates at four Ethiopian Tertiary Hospitals.

Analysis of AMR gene prevalence across the four study hospitals revealed distinct resistance profiles and variation in the distribution of key AMR genes (Table 2 and Figure 3). The four AMR genes with high prevalence and consistent presence across all hospitals were *bla*_CTX − M−15_ 97%(*n* = 94/97), *oqxA* 92%(*n* = 89/97), *fosA* 90%(*n* = 87/97), and *bla*_TEM − 1_ 77%(*n* = 75/97). In DCSH, Northern Ethiopia, there was a high prevalence of the phenicol/quinolone resistance gene *oqxA* 100%(*n* = 10/10), *dfrA14* 90%(*n* = 8/10) *aph(6)-Id* 80%(*n* = 8/10), *aph(3*″*)-Ib* 80%(*n* = 8/10), and *catA2* 60%(*n* = 6/10), along with several other AMR genes (Table 2 and Figure 3). In HUCSH, Southern Ethiopia, there were high AMR gene rates of phenicol/quinolone resistance gene *oqxA* 95%(*n* = 19/20), sulfonamide resistance gene *sul2* 90%(*n* = 18/20), aminoglycoside resistance genes *aph(6)-Id* 85%(*n* = 17/20) and *aph(3*″*)-Ib* 85% (*n* = 17/20; Table 2 and Figure 3). In central Ethiopia, TASH exhibited a diverse AMR gene profile, with the most prevalent being *bla*_CTX − M−15_ 95%(*n* = 35/37), *fosA* 92%(*n* = 34/37), and *oqxA* 95%(*n* = 32/37), while Y12HMC showed the highest prevalence of *fosA* 97%(*n* = 29/30), *bla*_CTX − M−15_ 97%(*n* = 29/30), and *oqxA* 93%(*n* = 28/30). The clustering analysis showed that HUCSH from southern Ethiopia and Y12HMC from central Ethiopia clustered together, with a higher prevalence of ESBL and other specific AMR genes (Figure 3).

### Genetic diversity of *K. pneumoniae* isolates and minimum spanning tree (MST) analysis

**Figure 4:**
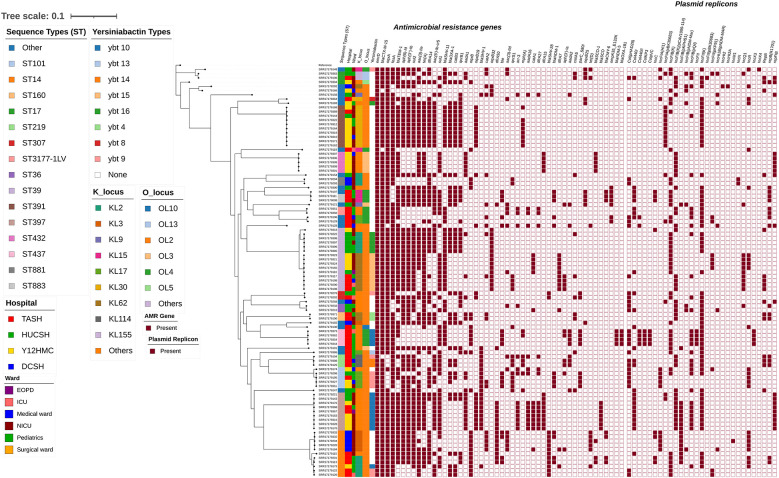
Maximum-likelihood phylogenetic tree of 97 *K. pneumoniae* isolates collected from four Ethiopian Tertiary Hospitals, reconstructed using IQ-TREE v3.0.1 under the GTR+G substitution model with 1,000 ultrafast bootstrap replicates and visualized. The tree is annotated with multilayered metadata, including sequence type (ST), hospital of origin, ward, *yersiniabactin* virulence, capsule (K-locus), lipopolysaccharide (O-locus), AMR genes, and plasmid replicon type.

**Figure 5:**
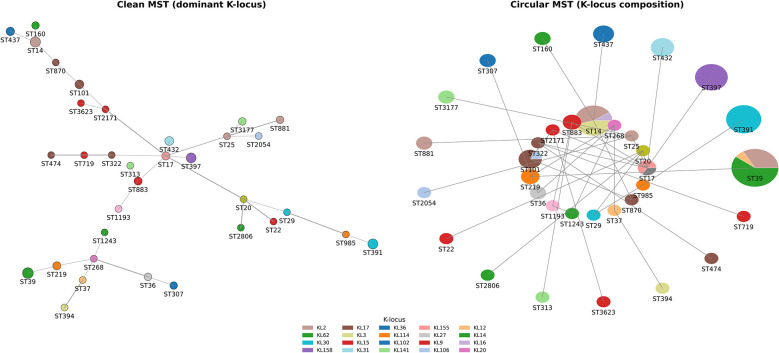
Minimum spanning tree of *K. pneumoniae* isolates illustrating the population structure and capsule (K-locus) diversity among the isolates. **(Left)** a clean MST with nodes colored by the dominant K-locus type for each sequence type (ST); node size is proportional to the number of isolates in each ST. Edges represent allelic distances between STs. **(Right)** circular MST with pie chart nodes showing the distribution of K-locus types within each ST. Node size reflects the number of isolates per ST, and edge weights denote allelic distances.

The maximum-likelihood phylogenetic tree of 97 *K. pneumoniae* isolates was annotated, revealing phylogenetically diverse clones circulating across the four hospitals (Figure 4). A minimum spanning tree (MST) was generated from the multi-locus sequence typing (MLST) profiles of these 97 isolates (Figure 4). The population structure of these isolates is polyphyletic, comprising 35 distinct sequence types (STs), indicating that no single outbreak strain dominated; instead, multiple expanding clones were involved (Figure 4 and Table 3). The most common clone was ST39 (*n* = 15), followed by ST14 (*n* = 11), ST391 (*n* = 10), and ST397 (*n* = 9; Table 3), each forming separate branches in the MST diagram (see Figure 5, clean MST). These sequence types were found across various wards of the four hospitals (Figure 4).

**Table 3:** Frequency and distribution of *K. pneumoniae* sequence types detected at four Ethiopian Tertiary Hospitals.

Sequence types (STs)	Overall frequency (*N* = 97) *n*(%)	TASH (*N* = 37) *n*(%)	Y12HMC (*N* = 30) *n*(%)	HUCSH (*N* = 20) *n*(%)	DCSH (*N* = 10) *n*(%)
ST39	15(16)	4(11)	5(17)	6(30)	–
ST14	11(11)	5(14)	1(3)	–	5(50)
ST391	10(10)	1(3)	8(27)	1(5)	–
ST397	9(9)	2(5)	5(17)	2(10)	–
ST101	5(5)	1(3)	3(10)	1(5)	–
ST432	5(5)	–	5(17)	–	–
ST437	4(4)	4(11)	–	–	–
ST17	3(3)	1(3)	1(3)	1(5)	–
ST219	3(3)	3(8)	–	–	–
ST36	2(2)	–	–	2(10)	–
ST160	2(2)	2(5)	–	–	–
ST307	2(2)	1(3)	–	1(5)	–
ST881	2(2)	–	–	–	2(20)
ST883	2(2)	2(5)	–	–	–
ST3177-1LV	2(2)	2(5)	–	–	–
ST20	1(1)	–	1(3)	–	–
ST22	1(1)	–	–	1(5)	–
ST25	1(1)	–	–	1(5)	–
ST29	1(1)	1(3)	–	–	–
ST37	1(1)	1(3)	–	–	–
ST268	1(1)	–	–	–	1(10)
ST313	1(1)	–	–	1(5)	–
ST322	1(1)	1(3)	–	–	–
ST394	1(1)	–	–	–	1(10)
ST474	1(1)	1(3)	–	–	–
ST719	1(1)	–	–	1(5)	–
ST870	1(1)	1(3)	–	–	–
ST883-1LV	1(1)	1(3)	–	–	–
ST985	1(1)	–	1(3)	–	–
ST1193	1(1)	–	–	1(5)	–
ST1243	1(1)	1(3)	–	–	–
ST2054	1(1)	1(3)	–	–	–
ST2171	1(1)	–	–	1(5)	–
ST2806	1(1)	–	–	–	1(10)
ST3623	1(1)	1(3)	–	–	–

The distribution of sequence types varied across hospitals: TASH had the highest diversity, with 21 unique STs among 37 isolates, followed by HUCSH (13 STs/20 isolates), Y12HMC (9 STs/30 isolates), and DCSH (5 STs/10 isolates; Table 3). In central Ethiopia, clones ST14, ST39, and ST437 were frequently identified at TASH, whereas at Y12HMC, ST391, ST39, ST397, and ST432 were more common. Conversely, at HUCSH in southern Ethiopia, ST39 was the most prevalent; however, no ST39 cases were found at DCSH. Instead, at DCSH in northern Ethiopia, ST14 was the dominant sequence type at 50%(*n* = 5/10), while the remaining cases were ST268, ST394, ST881, and ST2806 (Table 3). All hospitals harbored one or more well-known high-risk clones, such as ST14, ST101, ST307, ST17, ST25, and ST37 (Table 3), suggesting the potential spread or introduction of epidemiologically important lineages into the healthcare system.

β-lactam resistance genes were mostly conserved across different sequence types (STs; Figure 4 and Supplementary Table S1). Nearly all sequence types (*n* = 94) carried *bla*_CTX − M−15_, with three rare exceptions identified at two hospitals in central Ethiopia: ST219 and ST3623 at TASH and ST20 at Y12HMC. Similarly, the *bla*_TEM − 1_ gene was mainly found in ST14, ST17, ST39, ST391, ST397, and ST432. While *bla*_SHV − 11_ was present in some types, such as ST17, ST39, ST391, and ST437, and in a few rarely detected other types, it was absent in most, including ST14, ST219, ST397, and ST432 (see Figure 4). Additionally, *bla*_NDM − 1_ was found in ST14, ST101, ST322, ST883, and ST2054, while *bla*_NDM − 5_ was found exclusively in ST437 (Figure 4 and Supplementary Table S1).

### Clonal clusters of *K. pneumoniae* both within and between hospitals

**Figure 6:**
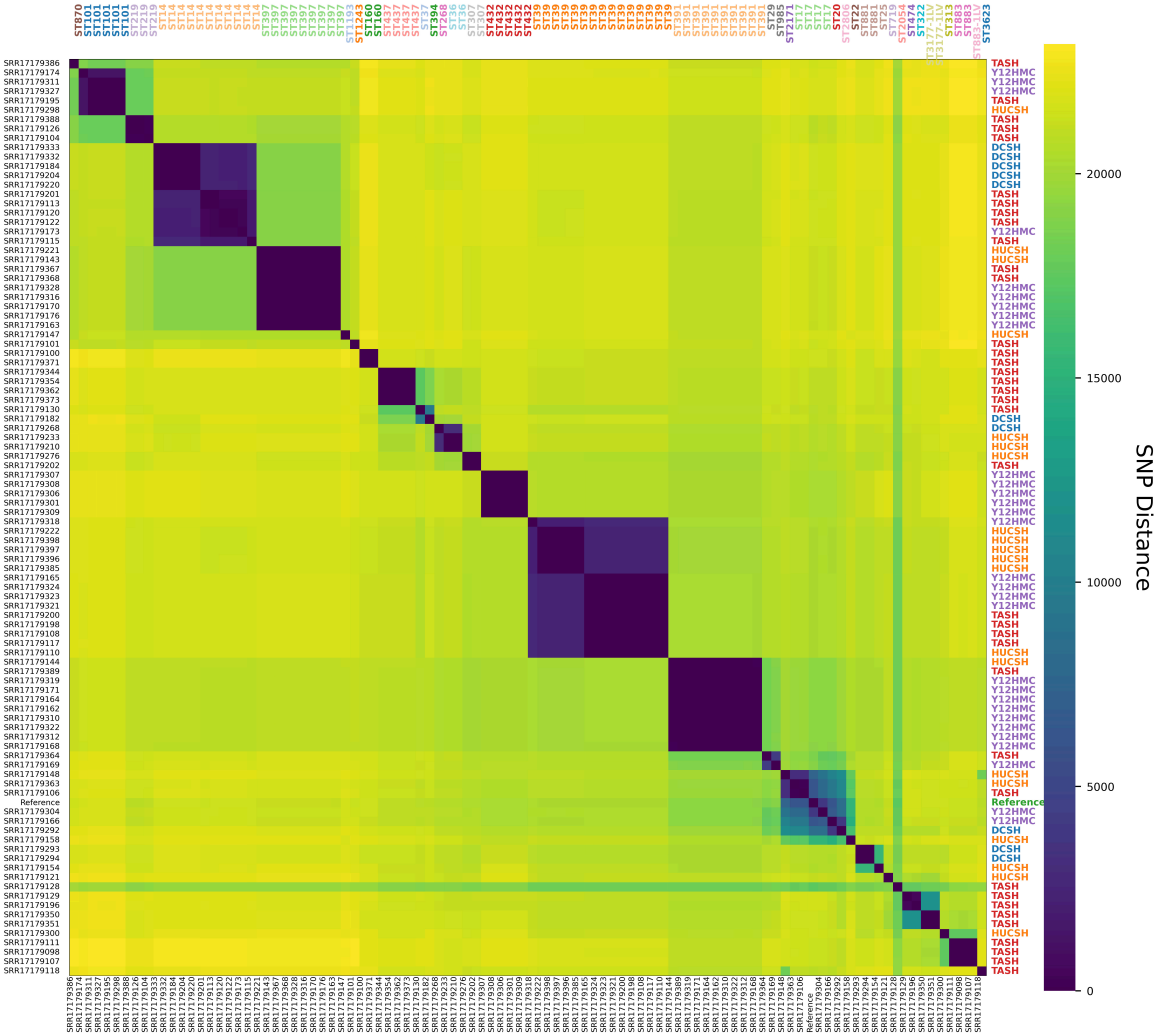
Hierarchical clustering of *K. pneumoniae* clinical isolates based on pairwise single-nucleotide polymorphism (SNP) distances: Heatmap displays pairwise SNP distances between 97 *K. pneumoniae* isolates [including reference strain (NZ_CP009461.1), ST17]. Sample IDs are shown on the *x*-axis and to the left (*y*-axis). Sequence types (STs) are indicated at the top of the heatmap (colored by ST type), and hospitals are shown to the right (colored by hospital). The viridis colormap ranges from 0 (minimum, dark purple) to 23,172 (maximum, yellow) for SNP distances. Clusters of closely related isolates suggest potential transmission events or clonal expansion within and between hospitals.

The SNP distance matrix revealed distinct clusters of genetically similar *K. pneumoniae* isolates (Figure 6). Hierarchical clustering grouped isolates with minimal SNP differences, often corresponding to shared hospital origin or identical ST type. The majority of isolates within the same cluster shared the same ST, confirming the concordance between SNP-based phylogeny and MLST typing. Many isolate pairs differed by fewer than 100 core-genome SNPs. These closely related pairs often, but not always, originated from the same hospital (Figure 6).

For instance, several 0–1 SNP pairs were found within the large ST391, and all ST432 (*n* = 5) clusters with 0–1 SNP differences included only isolates from Y12HMC (Figure 6). Several tight clusters ( ≤ 20 SNPs) were also observed within individual hospitals, suggesting potential nosocomial transmission or clonal expansion. Notably, ST437 was exclusively found in TASH (*n* = 4) with low diversity (2–6 SNPs), suggesting a recent, localized outbreak at the hospital. Conversely, cross-hospital clusters were identified, suggesting potential inter-facility spread (Figure 6). For example, ST39 clusters exhibited a broader range of SNP distances (0–48 SNPs) across the three hospitals in central and southern Ethiopia (TASH, Y12HMC, and HUCSH), with no cases at DSCH in the northern region. An additional example was a low diversity ST14 cluster that was primarily in TASH and DCSH (Figure 6). The cross-hospital clusters reflected higher genetic diversity than clusters confined to a single hospital. Likewise, the ST101 lineage encompassed closely related isolates from TASH, Y12HMC, and HUCSH (Figure 6).

### Virulence factors

The capsular (K-locus) and O-locus exhibited significant variation, with KL62, KL66, and KL2 being the most common, while O1/O2 and O3 were the main O-loci (Figures 4, 5). Mapping the capsular (K) loci to sequence types revealed two distinct evolutionary patterns: (1) sequence type conserved capsular loci, such as ST391 and ST397, showed complete consistency, being exclusively associated with KL30 and KL158, respectively, and (2) the most common sequence type displayed notable capsular diversity, exemplified by ST39 isolates, which included KL62, KL2, and KL51, and ST14 containing KL2, KL3, and KL16 (Figure 5 circular MST). Other virulence factors included *Yersiniabactin* (*ybt*), detected in 44 of 97 *K. pneumoniae* strains (45.4%; Figure 4). The yersiniabactin types identified were *ybt4, ybt8, ybt9, ybt10, ybt13, ybt14, ybt15*, and *ybt16*. These virulence genes were not found on plasmids, indicating that the virulence factors in this dataset were likely chromosomally encoded or located on contigs lacking identifiable plasmid replicons (Figure 4).

### Plasmid replicon diversity

All 97 *K. pneumoniae* isolates possessed a plasmid replicon and showed diversity (Figure 4 and Table 4). They belonged to groups such as Col, ColKP, ColpVC, IncF, IncHI, IncM, IncN, IncQ, IncR, and IncX (Table 4), which could contribute to the spread of antimicrobial resistance. The majority of the isolates carried plasmid replicons from the broad IncF family: IncFIB was present in 87.6%(*n* = 85/97) of isolates and IncFII in 76.3%(*n* = 74/97), and the specific plasmid replicon IncFII(K) was found in 69.1%(*n* = 67/97; Table 4). Col plasmid groups were also common, carried by 48.5%(*n* = 47/97) of isolates, while other prevalent replicons included IncFIA 29.9%(*n* = 29/97), IncR 24.7%(*n* = 24/97), repFIB 20.6%(*n* = 2=/97), repB 17.5%(*n* = 17/97), and RepB 16.5%(*n* = 16/97; Table 4). The plasmid profile was dominated by a limited number of high-frequency replicon types, with IncFIB(K) 43.3%(*n* = 42/97), IncFIB(K)(pCAV1099-114) 38.1%(*n* = 37/97), IncFIB(pQil) 32.0%(*n* = 31/97), Col(pHAD28) 27.8%(*n* = 27/97), and Col440I 26.8%(*n* = 26/97) being the most common specific types identified (Table 4). Among these, the IncF group was the most widely distributed across all hospitals. The IncFII(K) replicon type was highly prevalent in HUCSH 90.0%(*n* = 18/20) compared with other sites, whereas DCSH isolates showed the highest proportions of IncR 60.0%(*n* = 6/10) and IncFIA(HI1) 50.0%(*n* = 5/10). TASH isolates were notable for carrying the ColKP3 10.8%(*n* = 4/37) and ColpVC 10.8%(*n* = 4/37) replicons, and was the only hospital at which the carbapenemase-associated IncX3 replicon was detected 10.8%(*n* = 4/97; Table 4 and Figure 4).

**Table 4:** Overall frequency and distribution of plasmid replicons found in *K. pneumoniae* isolated from sepsis patients at four Ethiopian Tertiary Hospitals.

Plasmid group	Overall plasmid group (*N* = 97) *n*(%)	Plasmid type	Overall plasmid type (*N* = 97) *n*(%)	TASH (*N* = 37) *n*(%)	Y12HMC (*N* = 30) *n*(%)	HUCSH (*N* = 20) *n*(%)	DCSH (*N* = 10) *n*(%)
IncFIB	85(87.6)	IncFIB(K)	42(43.3)	20(54.1)	8(26.7)	11(55.0)	3(30.0)
		IncFIB(K)(pCAV1099-114)	37(38.1)	15(40.5)	12(40.0)	8(40.0)	2(20.0)
		IncFIB(pKPHS1)	17(17.5)	6(16.2)	5(16.7)	1(5.0)	5(50.0)
		IncFIB(pNDM-Mar)	8(8.2)	–	2(6.7)	4(20.0)	2(20.0)
		IncFIB(pQil)	31(32.0)	16(43.2)	7(23.3)	7(35.0)	1(10.0)
IncFII	74(76.3)	IncFII	4(4.1)	4(10.8)	–	–	–
		IncFII(K)	67(69.1)	29(78.4)	17(56.7)	18(90.0)	3(30.0)
		IncFII(pBK30683)	1(1.0)	1(2.7)	–	–	–
		IncFII(pKP91)	10(10.3)	4(10.8)	5(16.7)	1(5.0)	–
Col	47(48.5)	Col(pHAD28)	27(27.8)	17(45.9)	3(10.0)	4(20.0)	3(30.0)
		Col440I	26(26.8)	14(37.8)	5(16.7)	7(35.0)	–
		Col440II	6(6.2)	6(16.2)	–	–	–
IncFIA	29(29.9)	IncFIA(HI1)	13(13.4)	3(8.1)	3(10.0)	2(10.0)	5(50.0)
		IncFIA(pBK30683)	16(16.5)	2(5.4)	8(26.7)	5(25.0)	1(10.0)
IncR	24(24.7)	IncR	24(24.7)	6(16.2)	9(30.0)	3(15.0)	6(60.0)
repFIB	20(20.6)	repFIB	20(20.6)	4(10.8)	11(36.7)	4(20.0)	1(10.0)
repB	17(17.5)	repB(R1701)	17(17.5)	14(37.8)	1(3.3)	2(10.0)	–
RepB	16(16.5)	repB	16(16.5)	10(27.0)	3(10.0)	2(10.0)	1(10.0)
IncQ	12(12.4)	IncQ1	12(12.4)	4(10.8)	4(13.3)	4(20.0)	–
IncC	8(8.2)	IncC	8(8.2)	5(13.5)	1(3.3)	–	2(20.0)
IncHI	7(7.2)	IncHI1B(pNDM-MAR)	6(6.2)	1(2.7)	1(3.3)	3(15.0)	1(10.0)
		IncHI2	1(1.0)	–	–	–	1(10.0)
		IncHI2A	1(1.0)	–	–	–	1(10.0)
ColKP	4(4.1)	ColKP3	4(4.1)	4(10.8)	–	–	–
ColpVC	4(4.1)	ColpVC	4(4.1)	4(10.8)	–	–	–
IncX	5(5.2)	IncX3	4(4.1)	4(10.8)	–	–	–
		IncX4	1(1.0)	–	–	1(5.0)	–
IncN	2(2.1)	IncN	2(2.1)	–	–	–	2(20.0)
IncM	1(1.0)	IncM1	1(1.0)	–	–	–	1(10.0)

Several sequence types shared common plasmid backbones, suggesting possible horizontal gene transfer and conserved resistance platforms. Notably, IncFII(K) and IncFIB(K) were widespread across multiple STs, including ST14, ST17, ST39, ST101, ST219, ST391, and ST397. Variants of IncFIB, such as IncFIB(pKPHS1) and IncFIB(pQil), were also frequently found in ST14, ST101, ST391, ST397, ST432, and ST437. Additionally, Col-type plasmids (such as Col440I) and repFIB were detected in different STs, especially ST391, ST397, and ST432. These overlaps emphasize a shared plasmid pool that facilitates the spread of resistance despite genetic diversity among sequence types (Figure 4 and Supplementary Table S1).

### Plasmid-AMR-gene associations among *K. pneumoniae*

**Figure 7:**
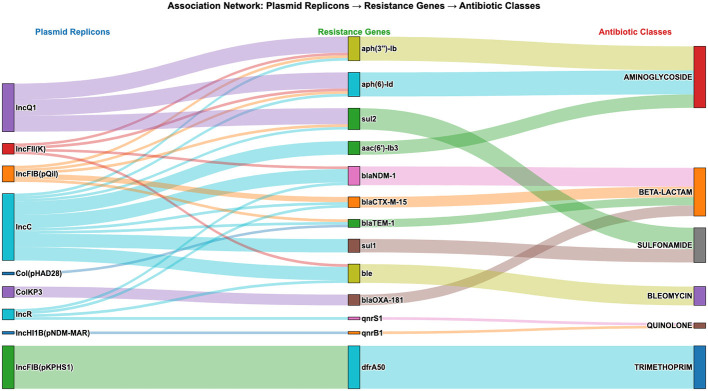
Sankey diagram of associations between plasmid replicons and antibiotic resistance determinants. The visualization shows the flow of associations from plasmid replicon types **(Left)** to specific AMR genes **(Center)** and to antibiotic classes **(Right)**. Link widths are proportional to the frequency of associations.

Analysis of plasmid-AMR gene integration uncovered a complex network linking plasmid replicons to AMR determinants (Figure 7). There were 79 associations between plasmids and AMR genes across 37 samples, meaning these samples contained plasmid-borne AMR genes. Additionally, 9 plasmid replicon types carried AMR genes, and a total of 13 unique AMR genes were present on plasmids. A Sankey diagram (Figure 7) illustrates 79 unique connections involving nine plasmid replicon types, six antibiotic classes, and 13 resistance genes. The diagram depicts the flow of associations from plasmid replicons to resistance genes and then to specific antibiotic classes, with link widths proportional to the frequency of these associations.

IncC and IncFIB(pKPHS1) plasmids were the most frequently detected replicon groups, accounting for 31.6% (*n* = 25/79) and 20.3% (*n* = 16/79) of plasmid-associated AMR events, respectively. The Sankey diagram (Figure 7) shows that IncC has the broadest range of AMR genes, conferring resistance to four antibiotic classes (aminoglycosides, beta-lactams, bleomycin, and sulfonamides), and it includes nine distinct resistance genes. In contrast, IncQ1 plasmids exhibited strong linkage to aminoglycoside and sulfonamide resistance, while IncFIB(pKPHS1) plasmids were solely associated with trimethoprim resistance via the *dfrA50* gene (Figure 7).

Many β-lactamase (*bla*) genes were mostly discovered on plasmids (see Figures 4, 7 and Supplementary Table S1). Notably, *bla*_CTX − M−15_ (an ESBL gene) and *bla*_TEM − 1_ (a common β-lactamase gene) were associated with the IncC plasmid replicon (refer to Figure 7). A total of 11 links with carbapenemase resistance genes were identified. The *bla*_NDM − 1_ gene was predominantly found on three plasmid types: IncC, IncFII(K), and IncR. Meanwhile, *bla*_OXA − 181_ was located on ColKP3. Overall, 11 connections between different plasmid replicons and carbapenemase resistance genes were observed (Figures 4, 7, and Supplementary Table S1).

## Discussion

*K. pneumoniae* resistant to third-generation cephalosporins due to ESBLs and to carbapenems because of carbapenemases is classified as a top priority by the WHO (Organization, 2024). Multiple studies from different countries have shown that ESBLs and carbapenemase-producing *K. pneumoniae* are spread worldwide and pose a serious public health threat (Hussain et al., 2023; Sedrakyan et al., 2025; Lopes et al., 2025; Nicitra et al., 2025; Rojas et al., 2017; Sands et al., 2021; Labi et al., 2020; Wu et al., 2019; Azra et al., 2025; Mohamed et al., 2025; Maguire et al., 2025; Fassbind et al., 2025). A 2019 report (Ikuta et al., 2022) indicated that multidrug-resistant *K. pneumoniae* is associated with high mortality rates in sepsis patients. Using whole-genome sequencing, this study examined 97 *K. pneumoniae* strains isolated from 1,416 sepsis patients across four hospitals in central, southern, and northern Ethiopia to determine sequence types, resistomes, plasmid replicons, virulence factors, and other genomic characteristics. At each study site, *K. pneumoniae* was identified as a significant causative agent of sepsis.

This study identified a wide array of phylogenetically diverse *K. pneumoniae* strains (Figure 4), with 35 distinct sequence types (STs; Table 3). Similarly, a prior study in western Ethiopia, which used a range of clinical samples beyond blood, also found multiple *K. pneumoniae* clones in the hospital; 40 STs were identified at a single hospital (Sewunet et al., 2021). In the current study, the most common STs were ST39 (*n* = 15), ST14 (*n* = 11), ST391 (*n* = 10), and ST397 (*n* = 9), while ST101 (*n* = 5), ST17 (*n* = 3), and ST307 (*n* = 2) were identified as internationally high-risk clones (Table 3). The detection of diverse *K. pneumoniae* clones across multiple hospitals in various regions of the country demonstrated the nationwide burden of this pathogen.

The detection of multiple isolate pairs with few SNP differences (Figure 6), especially from the same hospital, strongly suggested recent clonal transmission. Further epidemiological investigation is necessary to identify common wards, times, or patient links. A hospital-specific intervention strategy could help prevent further transmission. Conversely, the identification of closely related clones, such as ST39 and ST101 (Figures 4, 6), across multiple hospitals highlights inter-hospital spread of well-adapted *K. pneumoniae* ST strains within the country's healthcare network. Transmission routes may include patient transfers, shared staff, or community reservoirs. Including previous work in western Ethiopia (Sewunet et al., 2021), the widespread ST39 cluster across different hospitals suggests it may be a regional clone, warranting coordinated surveillance and countrywide intervention policies and calling for further study.

All *K. pneumoniae* strains in this study carried multiple AMR genes (Table 2 and Figures 3, 4) for β-lactams, aminoglycosides, phenicol, trimethoprim, macrolides, quinolones, tetracyclines, and sulfonamides, with no isolates lacking AMR genes, suggesting a high level of antimicrobial resistance in the country, supporting previous reports (Sewunet et al., 2021; Tufa et al., 2022; Gebremeskel et al., 2023). Nearly all *K. pneumoniae* isolates (97%) possessed ESBL genes, predominantly *bla*_CTX − M−15_ (*n* = 94), along with various resistance genes spanning multiple antibiotic classes (Table 2 and Figure 4). The high prevalence of ESBLs among *K. pneumoniae* was consistent with studies conducted in numerous countries, including the previous work in western Ethiopia (Sedrakyan et al., 2025; Lopes et al., 2025; Sewunet et al., 2021; Sands et al., 2021; Labi et al., 2020; Hristea et al., 2015). Even more concerning, the detection of carbapenemase-encoding genes carried on plasmids (Figure 7), primarily *bla*_NDM − 1_, across all hospitals, and the detection of *bla*_NDM − 5_ and *bla*_OXA − 181_ at TASH, adds to the growing challenge of antimicrobial resistance in the country. Studies worldwide (Melinte et al., 2025; Nicitra et al., 2025; Qamar et al., 2025; Rojas et al., 2017; Kumwenda et al., 2021; Martínez-Álvarez et al., 2025) have documented the global dissemination of *bla*_NDM_ and *bla*_OXA_ carbapenemase variants. Colistin resistance caused by the *mcr* gene was rare, observed in only one isolate with *mcr 9.1*, which is significant for public health and indicates that colistin remains a useful drug; however, attention should be given to halt the occurrence and spread of these resistance genes.

It has been known that plasmids facilitate the dissemination of AMR genes (Sedrakyan et al., 2025; Qamar et al., 2025; Rozwandowicz et al., 2018; Cerdeira et al., 2019), and this study identified several AMR genes associated with different antibiotic classes (Figure 7), each linked to distinct plasmid replicons. Similar to previous studies (Nguyen et al., 2024; Qamar et al., 2025; Rozwandowicz et al., 2018; Cerdeira et al., 2019), the most common plasmid replicons identified in this study were Col, ColKP, ColpVC, IncF, IncHI, IncM, IncN, IncQ, IncR, and IncX (Table 4). In the current study, analysis of plasmids carrying AMR genes revealed a complex network linking these replicons to various AMR determinants (Figure 7). IncC was the primary plasmid replicon carrying multiple AMR genes, including ESBLs (*bla*_CTX − M−15_ and *bla*_TEM − 1_) and a carbapenemase (*bla*_NDM − 1_; Figure 7), as previously reported (Qamar et al., 2025). These findings support responsible authorities in creating and enforcing effective antimicrobial stewardship programs. Among the virulence factors (Figure 4), *yersiniabactin* types (*ybt4, ytb8, ybt9, ybt10, ybt13, ybt14, ytb15*, and *ybt16*) were found in 45% *of K. pneumoniae* isolates, as previously reported (Wisgrill et al., 2019). However, no other virulence factors were identified. On the other hand, variable capsular (K-type and O-locus) variants were observed (Figure 5), which could help elucidate the epidemiology and virulence of *K. pneumoniae*.

This genomic analysis of *K. pneumoniae* underscores the importance of strengthening infection prevention and control (IPC) alongside antimicrobial stewardship (AMS) programs. Improving these initiatives can lead to better sepsis management and help limit the spread of antimicrobial resistance (AMR), particularly in resource-constrained environments. Differences in sepsis severity and AMR patterns among hospitals indicate regional transmission and local circulation, so IPC and AMS strategies should be customized for each location and coordinated across healthcare facilities to achieve the best outcomes.

### Limitations of the study

The study's strengths include the identification and reporting of clonally diverse MDR *K. pneumoniae* strains from hospitals across various regions, using whole genome sequencing. However, it is limited by a lack of data on the risk factors and patient outcomes linked to these multidrug-resistant *K. pneumoniae* isolates.

### Conclusion

*Klebsiella pneumoniae* strains from sepsis patients in Ethiopia exhibited notable genomic diversity, circulating within and between hospitals in the central, southern, and northern regions. The widespread occurrence of diverse *K. pneumoniae* clones carrying plasmid-encoded ESBL genes, along with various other AMR genes targeting multiple antibiotic classes, poses a major public health threat. Moreover, the identification of carbapenemase-producing *K. pneumoniae* isolates is particularly worrisome. These findings highlight the urgent need to improve infection control and antimicrobial stewardship efforts nationwide.

## Data Availability

The data presented in the study are deposited in the National Center for Biotechnology Information repository with BioProject ID: PRJNA787062.
