# Supplementary material for: Genomic characterization of Klebsiella pneumoniae isolates from sepsis patients in Ethiopian tertiary hospitals: a multicenter cross-sectional study
Source: Front Microbiol. 2026 Apr 28;17:1775426. doi: 10.3389/fmicb.2026.1775426 (PMC13161078; doi:10.3389/fmicb.2026.1775426)
Supplement: Supplementary file 1 [file Table_1.docx]

**Supplementary Table S1. Distribution of *bla* genes and plasmid replicons by sequence type (ST). Each row is an ST group; values shown as % of isolates within that ST group carrying the gene/replicon.**

| **ST** | **n** | ***bla* genes detected (%, within ST group)** | **Replicons detected (%, within ST group)** |
| --- | --- | --- | --- |
| **ST39** | **15** | *bla*_CTX-M-15_ (100); *bla*_SHV-11_ (100); *bla*_TEM_ (100); *bla*_OXA-1_ (40); *bla*_OXA-10_ (13) | IncFIB(K) (67); IncQ1 (60); IncFII(K) (40); IncFIB(K)(pCAV1099-114) (33); RepB (33); IncR (27); IncFII(pKP91) (27); IncFIB(pQil) (13); Col(pHAD28) (7); Col440I (7); IncC (7) |
| **ST14** | **11** | *bla*_CTX-M-15_ (100); *bla*_SHV-60_ (100); *bla*_TEM-1_ (73); *bla*_CMY-6_ (36); *bla*_NDM-1_ (36); *bla*_OXA-1_ (36); *bla*_SCO_ (27); *bla*_TEM_ (18); *bla*_SHV-11_ (9); *bla*_SHV_C-112A_ (9) | IncFIB(pKPHS1) (73); IncR (73); IncFIB(K) (55); IncFIA(HI1) (46); IncC (36); IncFII(K) (36); IncFII(pKP91) (18); repB(R1701) (18); Col(pHAD28) (18); Col440I (9); IncFIB(pNDM-Mar) (9); IncFIB(pQil) (9); IncHI1B(pNDM-MAR) (9); IncM1 (9) |
| **ST391** | **10** | *bla*_CTX-M-15_ (100); *bla*_OXA-1_ (100); *bla*_SHV-11_ (100); *bla*_TEM-1_ (100) | IncFIA(pBK30683) (100); IncFII(K) (100); IncFIB(K) (10); IncFIB(pQil) (10) |
| **ST397** | **9** | *bla*_CTX-M-15_ (100); *bla*_SHV_ (100); *bla*_TEM-1_ (100); *bla*_OXA-181_ (78); *bla*_OXA-1_ (22) | Col440I (100); IncFIB(K)(pCAV1099-114) (100); IncFII(K) (100); repFIB (100); IncFIB(pKPHS1) (78); IncFIB(pQil) (78); IncFIA(pBK30683) (22.22) |
| **ST101** | **5** | *bla*_CTX-M-15_ (100); *bla*_NDM-1_ (80); *bla*_OXA-1_ (60); *bla*_SHV_ (100); *bla*_TEM-1_ (60); *bla*_OXA_ (40) | Col(pHAD28) (100); IncFIA(HI1) (100); IncR (100); IncFIB(K) (40); IncFIB(pQil) (40); IncFII(K) (40); IncFII(pKP91) (20) |
| **ST432** | **5** | *bla*_CTX-M-15_ (100); *bla*_SCO-1_ (100); *bla*_SHV28_ (100); *bla*_TEM-1_ (100) | IncFIB(K)(pCAV1099-114) (100); repFIB (100) |
| **ST437** | **4** | *bla*_CTX-M-15_ (100); *bla*_NDM-5_ (100); *bla*_OXA-10_ (100); *bla*_OXA-181_ (100); *bla*_SHV-11_ (100); *bla*_TEM-1_ (50) | Col(pHAD28) (100); Col440II (100); ColKP3 (100); ColpVC (100); IncFIB(K) (100); IncFIB(pQil) (100); IncFII (100); IncFII(K) (100); IncX3 (100) |
| **Others** |  | *bla*_CTX-M-15_ (92); *bla*_TEM-1_ (61); *bla*_SHV-11_ (37); *bla*_OXA-1_ (42); *bla*_TEM_ (3); *bla*_CMY-6_ (8); *bla*_CTX-M_ (3); *bla*_DHA-1_ (5); *bla*_NDM-1_ (13); *bla*_SHV_ (18); *bla*_SHV-110_ (3); *bla*_SHV-119_ (5); *bla*_SHV-187_ (11); *bla*_SHV-27_ (11); *bla*_SHV-28_ (5); *bla*_SHV-71_ (8); *bla*_SCO_ (0); *bla*_SCO-1_ (0); *bla*_OXA-10_ (11); *bla*_OXA-181_ (0); *bla*_SHV_C-112A_ (0) | IncFII(K) (84); IncFIB(K) (50); IncFIB(K)(pCAV1099-114) (47); Col(pHAD28) (40); Col440I (40); repB(R1701) (40); RepB (29); repFIB (16); IncHI1B(pNDM-MAR) (13); IncFIA(pBK30683) (11); IncFIB(pQil) (37); IncFIB(pNDM-Mar) (18); IncFII(pBK30683) (3); IncFII(pKP91) (8); IncC (8); IncFIA(HI1) (8); IncN (5); IncQ1 (8); IncR (18); IncHI2 (3); IncHI2A (3); Col440II (5); IncX4 (3) |

Footnote: "Others" includes ST391, ST397, ST432, ST437, ST101, ST219, ST17, ST883, ST881, ST307, ST3177-1LV, ST36, ST160, ST1243, ST1193, ST20, ST2054, ST322, ST313, ST2806, ST29, ST268, ST25, ST2171, ST22, ST394, ST37, ST3623, ST474, ST870, ST719, ST883-1LV, ST985.
